# A targeted PCR approach for the detection of IOLA in canine infectious respiratory disease samples during an atypical CIRD outbreak in winter 2023

**DOI:** 10.3389/fvets.2026.1849862

**Published:** 2026-06-26

**Authors:** Mahamudul Hasan, Erin E. Schirtzinger, Steven Stancic, Pedro Affonso, Andrea Lu, Jayme A. Souza-Neto, Katherine L. Klenda, Franco Matias Ferreyra, Lance W. Noll, Gregg A. Hanzlicek, Jamie Retallick, Laura C. Miller

**Affiliations:** 1 Department of Diagnostic Medicine/Pathobiology, College of Veterinary Medicine, Kansas State University, Manhattan, KS, United States; 2 Life Span Institute, University of Kansas, Lawrence, KS, United States; 3 Kansas State Veterinary Diagnostic Laboratory, College of Veterinary Medicine, Kansas State University, Manhattan, KS, United States

**Keywords:** canine, CIRD (canine infectious respiratory disease), dual-target PCR, IOLA (Infectious Organism Lurking in Human Airways), respiratory

## Abstract

**Background:**

During winter 2023, an atypical canine infectious respiratory disease (aCIRD) outbreak was associated with high case-fatality rates and poor antibiotic response. Preliminary metagenomics investigations claimed partial sequences resembling Infectious Organism Lurking in Human Airways (IOLA), a poorly characterized bacterium first described in humans, in canine respiratory samples. However, its detectability remained uncertain and required systematic molecular investigation.

**Methods:**

We screened 777 veterinarian-submitted canine respiratory samples from the United States using *16S* targeted sequencing for samples positive for Rickettsiales, the lowest taxonomic classification for IOLA. Samples containing sequencing reads classified as for Rickettsiales were tested by PCR assay targeting two IOLA genes (*16S rRNA* and *PrfA*). Assays were optimized at 58 °C with 500 nM primers, and products visualized by agarose gel and capillary electrophoresis. Analytical sensitivity was 10^4^ copies/μl and 10^4^ copies/μl for *16S* and PrfA assay, respectively. Candidate amplicons were verified by Sanger sequencing and BLAST analysis.

**Results:**

Of the 777 samples screened, 55 contained sequencing reads classified as Rickettsiales. Forty-five of the 55 samples were negative by *16S rRNA* PCR, while 10 samples produced amplicons near the expected size. The *PrfA* PCR assay was negative across all samples. Sequencing representative samples from those that produced *16S* amplicons confirmed nonspecific amplification. Therefore, all 55 samples were negative for IOLA.

**Conclusion:**

Dual-target PCR identified no evidence of IOLA in respiratory samples from the 2023 aCIRD outbreak. Non-specific amplification in the *16S* PCR assay highlights the need for multi-target validation in novel pathogen detection and supports prioritization of established CIRD pathogens over unverified organisms.

## 1 Introduction

Canine Infectious Respiratory Disease (CIRD) complex, commonly known as “kennel cough,” represents a major welfare issue for kennel facilities, pet owners, and veterinarians worldwide (1, 2). The causative agents of CIRD are highly contagious and spread through direct contact and via aerosols, especially in densely populated environments such as kennels, rehoming centers, shelters, dog parks, daycare facilities, and veterinary clinics (1, 3). Dogs with CIRD may develop clinical signs such as coughing, sneezing, nasal and ocular discharge, fever, lethargy, dyspnea, secondary lower respiratory tract infections, and sometimes fatal pneumonia (1, 4, 5). These symptoms can be the result of infection by viral and/or bacterial agents, such as *Bordetella bronchiseptica* (6), canine adenovirus type 2 (7), canine parainfluenza virus, canine herpesvirus type 1, and canine distemper virus (8). More recently, additional pathogens have been identified in association with symptomatic CIRD cases, including canine influenza virus, *Mycoplasma* species, *Streptococcus equi* subsp. *zooepidemicus*, canine respiratory coronavirus, and canine pneumovirus (8–11). The clinical complexity of CIRD is further increased due to the general nature of symptoms that can be the result of a single viral or bacterial infection, co-infection by several different agents or an opportunistic secondary bacterial infection, making the differentiation of the underlying etiology challenging (12).

Vaccination has become central to CIRD management and prevention (13); however, licensed vaccines are only available for a subset of CIRD agents (*Bordetella bronchiseptica*, canine adenovirus type 2, canine parainfluenza virus, canine distemper virus (CDV), and canine influenza viruses H3N8 and H3N2) (4, 8). For most of these pathogens, vaccination does not provide strong immunity. Although CDV vaccines are often considered comparatively more protective than several other CIRD vaccines, breakthrough disease can still occur, particularly when immunity is incomplete or when circulating CDV lineages differ from vaccine strains such as America-1. As a result, vaccinated dogs may still become subclinically infected and contribute to ongoing pathogen circulation and outbreak propagation. Compounding this issue, there are currently no licensed antiviral therapies for CIRD, and no formal guidelines supporting off-label antiviral use (8). Continued circulation of multiple respiratory pathogens under these conditions is suspected to contribute to the emergence of a newly described clinical presentation, referred to as atypical CIRD (aCIRD) (14, 15).

aCIRD gained national attention during the winter of 2023, when a sharp rise in cases of canine respiratory disease was reported in the United States (16). Many dogs developed persistent and chronic respiratory symptoms that rapidly progressed to pneumonia. Despite extensive testing, most cases were negative for known pathogens, and affected dogs responded poorly to antibiotic treatment, with mortality reaching approximately 9% (14, 15). These observations raised concerns about the possible emergence of a previously unrecognized organism associated with the outbreak. Investigators in New Hampshire sequenced respiratory samples from 70 dogs originating from New Hampshire, Rhode Island, and Massachusetts. Within the sequencing dataset, they found the ribosomal RNA sequence of a previously uncharacterized, unculturable, bacterial-like organism (17). Phylogenetic analysis showed that these sequences were classified as bacteria provisionally named Infectious Organism Lurking in Human Airways (IOLA) KY405, originally identified from a human bronchoalveolar lavage sample from a patient with chronic respiratory disease (18). However, the biological relevance of this sequence in canine disease remains uncertain, as the organism has not been isolated, its host range is unknown, and its presence in canine respiratory disease has not been independently verified.

The phylogenetic placement of IOLA is unclear as *16S rRNA* shows approximately 73% identity with known bacteria. Nearly half of the IOLA genomic coding sequences lack recognizable homology with genes of known function, while the remainder display similarities to genes within diverse bacterial, viral, and eukaryotic taxa. Ribosomal and housekeeping protein phylogenies place IOLA within the order Rickettsiales in the class Alphaproteobacteria (19). IOLA has an extremely reduced AT-rich genome and lacks many genes required for independent survival, indicating an obligate host-dependent lifestyle. The genome size and gene complement of IOLA suggests it may be analogous to insect endosymbionts (19). Despite this preliminary genomic characterization, significant knowledge gaps remain regarding IOLA’s host range, pathogenic potential, and detectability across different species. Currently, only a human-derived IOLA genomic reference (strain KY405) exists, and no confirmed animal-positive samples have been reported. However, detection of IOLA-like sequences during the 2023 aCIRD outbreak attracted broader scrutiny, including investigation guidance from the American Veterinary Medical Association. Within this broader outbreak context, our epidemiologic investigation identified unexpectedly high case-fatality rates, greater clinical severity, and poor antibiotic response in affected dogs (15). A parallel retrospective diagnostic surveillance analysis from the same period (unpublished data) further showed increased panel-negative submissions and reduced pathogen burden, particularly during colder months, raising the possibility that conventional multiplex qPCR targets did not fully account for all cases. Against this background, determining IOLA detectability in respiratory samples from affected dogs represented a critical step in evaluating its potential relevance to aCIRD. We therefore investigated a large cohort of suspected cases using IOLA-specific molecular assays, hypothesizing that if IOLA contributed to the outbreak, its DNA would be detectable in canine respiratory samples.

## 2 Methods

### 2.1 Sample collection and processing

Nasal, nasopharyngeal, and pharyngeal swabs from suspected CIRD cases submitted to the Kansas State Veterinary Diagnostic Laboratory between November 2023 and March 2024 were offered free bacterial *16S rRNA* amplicon sequencing. All samples were veterinarian-referred, included relevant patient information and owner consent to use the samples for research. During this period, 777 swab samples were received. Submitted specimens consisted of synthetic swabs with plastic shafts, including nylon flocked, Dacron, or rayon swabs; cotton-tipped swabs and wooden-shaft swabs were not received. Most swabs were submitted in transport medium, including Copan ESwab medium or general-purpose swab transport media such as liquid Amies or Stuart medium. A subset of samples was submitted as dry swabs; in these cases, 300–500 μL of Dulbecco’s Modified Eagle Medium was added by the laboratory to rehydrate the swabs.

### 2.2 DNA extraction and quantification

DNA was extracted from a maximum 400 μL of each sample using the MagMAX™ viral/pathogen nucleic acid isolation kit on a KingFisher™ flex magnetic particle processor, following the manufacturer’s protocol. DNA was eluted in 90 μL of elution solution. DNA concentrations were measured using the qubit 1X dsDNA HS assay kit according to manufacturer’s instructions. DNA was diluted to 5 ng/μL prior to amplification and library preparation.

### 2.3 Next generation sequencing

Samples were prepared following the Illumina *16S* Metagenomic Sequencing Protocol (20). Briefly, the V3–V4 region of *16S* rRNA was amplified using primers from Klindworth et al. (21) containing Illumina 5′ and 3′ overhang adapter sequences and 12.5 ng or 2.5 μL of DNA. Amplicons were purified with AMPure XP Beads into 50 μL 10 mM Tris, pH 8.5. 5 μL of the purified amplicons were subjected to a second PCR for dual indexing using the Nextera XT Index Kit initially for a few samples but for the vast majority of the samples, we used the IDT for Illumina DNA/RNA UD Indexes as it is compatible with this protocol. Indexed samples were quantified using the Qubit 1X dsDNA High Sensitivity (HS) Assay Kit. All libraries were individually quantified using the Qubit fluorometer. Rather than normalizing libraries to a uniform concentration prior to pooling as stated in the protocol, we generated the final sequencing pool by adding each library at a volume proportional to its measured concentration and the desired read depth. This approach allowed us to accommodate a variety of concentrations as some were too low to dilute to the target 4 nM. 5 microliters of each indexed sample was pooled, denatured and percentage of PhiX we spiked in varied across runs from 5 to 30% as an internal control. Sequencing was performed on the Illumina MiSeq platform using the MiSeq Reagent v3, 2×300 bp kit. Reads were demultiplexed using the Local Run Manager Generate FASTQ Analysis Module, and taxonomic classification was performed in QIAGEN CLC Genomics Workbench. Reads were trimmed and merged using default paired-end overlap settings and clustered into OTUs at 97% similarity. Taxonomic assignment was performed using the SILVA database, with sequences classified up to the order level based on CLC’s default k-mer–based matching parameters. Samples containing reads classified to the order Rickettsiales were selected for subsequent IOLA-specific PCR analysis. *16S* targeted sequencing was used solely as a sample selection filter and not as the primary detection method; the inherent limitations of partial *16S rRNA* gene analysis for species-level resolution of novel organisms have been well documented (22).

### 2.4 IOLA-specific PCR assay development

#### 2.4.1 Primer design

Previously published primers targeting the IOLA *16S rRNA* gene (19, 23) were evaluated and found to exhibit sequence homology with non-IOLA bacterial species in BLAST searches against the GenBank database. To ensure specific detection of IOLA, we focused on two genes in the IOLA genome: *16S rRNA* and peptide chain release factor 1 (*PrfA*). As canine-specific IOLA sequences are absent from public databases, PCR-based detection strategies must rely on primers designed from human-derived sequences. Thus, all primer sequences used in this study were designed from the IOLA genome originally characterized from a human respiratory specimen (GenBank: LC435447 and BCL65639). Primer candidates were generated using the PrimerQuest Tool (https://www.idtdna.com/pages/tools/primerquest) with standard parameters for primer length (18–25 nucleotides), GC content (35–60%), and melting temperature (Tm range 55–65 °C). Secondary structures and dimerization were evaluated using OligoAnalyzer (https://www.idtdna.com/calc/analyzer) and AutoDimer (24). Primer specificity was assessed using NCBI Primer-BLAST (https://www.ncbi.nlm.nih.gov/tools/primer-blast). For each gene, the primer pair showing optimal predicted performance and fewer off-target matches was selected. Final primer sequences and expected amplicon sizes are listed in Table 1.

**Table 1:** Primers characteristics for IOLA *16S rRNA* and IOLA *PrfA* genes.

Gene	Primer sequence	Expected amplicon size	Length	Tm (°C)	GC%
IOLA *16S rRNA*	Forward Primer: GGGCGAAGGCGATTAACTAT	92	20	62	50
	Reverse Primer: TTACAGCGTGGATTACAAGGG		21	62	47.6
IOLA *PrfA*	Forward Primer: GATTTCTTTCTTGCTGTGATAC	120	22	58	36.4
	Reverse Primer: GGGAAACATTTAAAGCATCAG		21	58	38.1

#### 2.4.2 Positive control development

##### 2.4.2.1 Synthetic control design

Positive control plasmids were synthesized by IDT. The amplified target sequences were extended by adding 25 nucleotides to both the 5′ and 3′ ends of each target gene’s amplicon. Positive control sequences are listed in Supplementary Data.

##### 2.4.2.2 Bacterial transformation, plasmid preparation and storage

Plasmids were transformed into chemically competent *Escherichia coli* Top10 cells (ThermoFisher Scientific), plated on LB agar plates with 50 mg/mL kanamycin. Transformed colonies were picked from plates and grown overnight in LB with kanamycin (50 mg/mL). Plasmid DNA was extracted using the ZymoPURE II Plasmid Maxiprep Kit following the manufacturer’s protocol. Purified plasmids were submitted to Azenta Life Sciences for Sanger sequencing to confirm the sequence insert in the plasmid (Supplementary Data). Plasmid concentration was determined with the Qubit dsDNA HS kit, copy number calculated and plasmids were diluted to 10^8^ copies/μL, aliquoted and stored at −20 °C until use.

#### 2.4.3 PCR optimization

##### 2.4.3.1 Assay optimization

To obtain optimal amplification of *16S rRNA* and *PrfA*, four annealing temperatures were tested: 50 °C, 55 °C, 58 °C, 60 °C. Each annealing temperature was evaluated in triplicate for each target. PCR reactions were prepared in 25 μL volumes containing Promega GoTaq Hot Star Green Master Mix (2X), 500 nM of forward and reverse primers and 5 μL plasmid DNA or nuclease-free water as a negative control and run on a Bio-Rad CFX96 Real-Time PCR System under the following conditions: 95 °C for 2 min; 40 cycles of 95 °C for 15 s, 50 °C, 55 °C, 58 °C or 60 °C for 15 s, and 72 °C for 10 s; with a final extension of 72 °C for 2 min. PCR products were visualized on a 3% agarose gel using SYBR Safe DNA Stain (Invitrogen) to determine amplicon size and quantity. The optimal annealing temperature was selected based on reproducible amplification across triplicate reactions, correct amplicon size, strongest consistent band intensity, minimal nonspecific amplification, and absence of amplification in the negative controls. Primer concentrations were optimized in a similar manner by testing 100 nM, 200 nM, 300 nM, 400 nM, and 500 nM of primers at the selected annealing temperature using the same PCR reaction mix and assay parameters as above. PCR products were visualized on a 3% agarose gel using SYBR Safe DNA Stain (Invitrogen) to determine amplicon size and quantity. The optimal concentration of primers was selected using the same criteria as the optimal annealing temperature. To confirm the specificity of the PCR assays, amplicons produced by the IOLA *16S rRNA* and *PrfA* assays were purified using the Zymo DNA Clean & Concentrator kit and submitted to Azenta Life Sciences for Sanger sequencing (Supplementary Figure 1).

##### 2.4.3.2 Limit of detection

To determine the analytical limit of detection (LOD), 10-fold serial dilutions of each plasmid (10^7^–10^0^ copies/μL) were amplified in duplicate under the optimized PCR conditions. PCR products were visualized using 3% agarose gel electrophoresis and by QIAxcel capillary electrophoresis using the standard QX 15 bp–3 kb alignment marker and 100 bp–2.5 kb size marker with AMP 420 method and injection time of 25 s. All other instrument parameters were kept at default settings. The baseline threshold of relative fluorescence units (RFU) was manually adjusted to 0.1 to distinguish true amplicons from background noise.

##### 2.4.3.3 PCR with clinical samples and PCR product analysis

Samples that contained V3–V4 *16S* sequences classified to the order Rickettsiales by sequencing were selected for IOLA detection by PCR. DNA extracts for the selected samples were acquired from KSVDL. Sample DNA concentration was quantified using the Qubit 1X dsDNA HS kit according to the manufacturer’s instructions (Invitrogen). Samples with high DNA concentrations were diluted to 5 ng/μL. IOLA *16S rRNA* and *PrfA* assays were run individually. PCR reactions contained Promega GoTaq Hot Star Green Master Mix (2X), selected concentration of forward and reverse primers and 5 μL of sample DNA or nuclease-free water as a negative control. Included in each run were serial dilutions of the appropriate positive control plasmid. Assays were run on a Bio-Rad CFX96 Real-Time PCR System under the following conditions: 95 °C for 2 min; 40 cycles of 95 °C for 15 s, selected annealing temperature for 15 s, and 72 °C for 10 s; with a final extension of 72 °C for 2 min. After amplification, PCR products were analyzed on the QIAxcel system using the criterion mentioned in method section 2.4.3.2. Results obtained from the QIAxcel for each sample included the size in bp of detected bands and the relative fluorescence units (RFU) of each band. A sample was classified as IOLA positive when both the *16S rRNA* and *PrfA* assays showed bands at approximately 92 and 120 bp, respectively and the RFU of each band was greater than 0.1. Several studies using the QIAxcel system set a 0.1 RFU threshold as the minimum cutoff to call amplicon peaks as positive (25, 26). This value reliably excludes instrument background noise, low-level artifacts. Besides, this cutoff value ensures only robust and true PCR products’ classification as positive (https://www.qiagen.com/us/resources/faq/1843). Samples were classified as IOLA negative when no bands were observed at 92 and 120 bp and/or RFU values were below 0.1. Samples that produced a PCR amplicon of the correct size in only the *16S rRNA or PrfA* assay were classified as ambiguous and further analyzed by Sanger sequencing and NCBI BLAST.

## 3 Results

### 3.1 Identification of Rickettsiales samples using NGS

Among the 777 canine respiratory samples analyzed by *16S* targeted sequencing, 635 contained reads from the bacterial class Alphaproteobacteria. Within this group, 55 samples contained reads classified to the order Rickettsiales. These 55 Rickettsiales samples were included for dual-target PCR of two IOLA genes (*16S rRNA* and *PrfA*). Demographic characteristics of these 55 samples are presented in Supplementary Figures 2, 3.

### 3.2 PCR assay optimization and validation

#### 3.2.1 Determination of optimal PCR conditions

Systematic evaluation of primer concentrations and thermal cycling parameters revealed optimal amplification at 500 nM concentration for both forward and reverse primers with an annealing temperature of 58 °C for both *16S rRNA* and *PrfA* assays (Supplementary Figures 4, 5). Across all PCR runs, positive-control plasmids consistently produced products at the expected sizes on 3% agarose gel. All no-template controls remained negative.

#### 3.2.2 Sensitivity analysis and limit of detection

**Figure 1:**
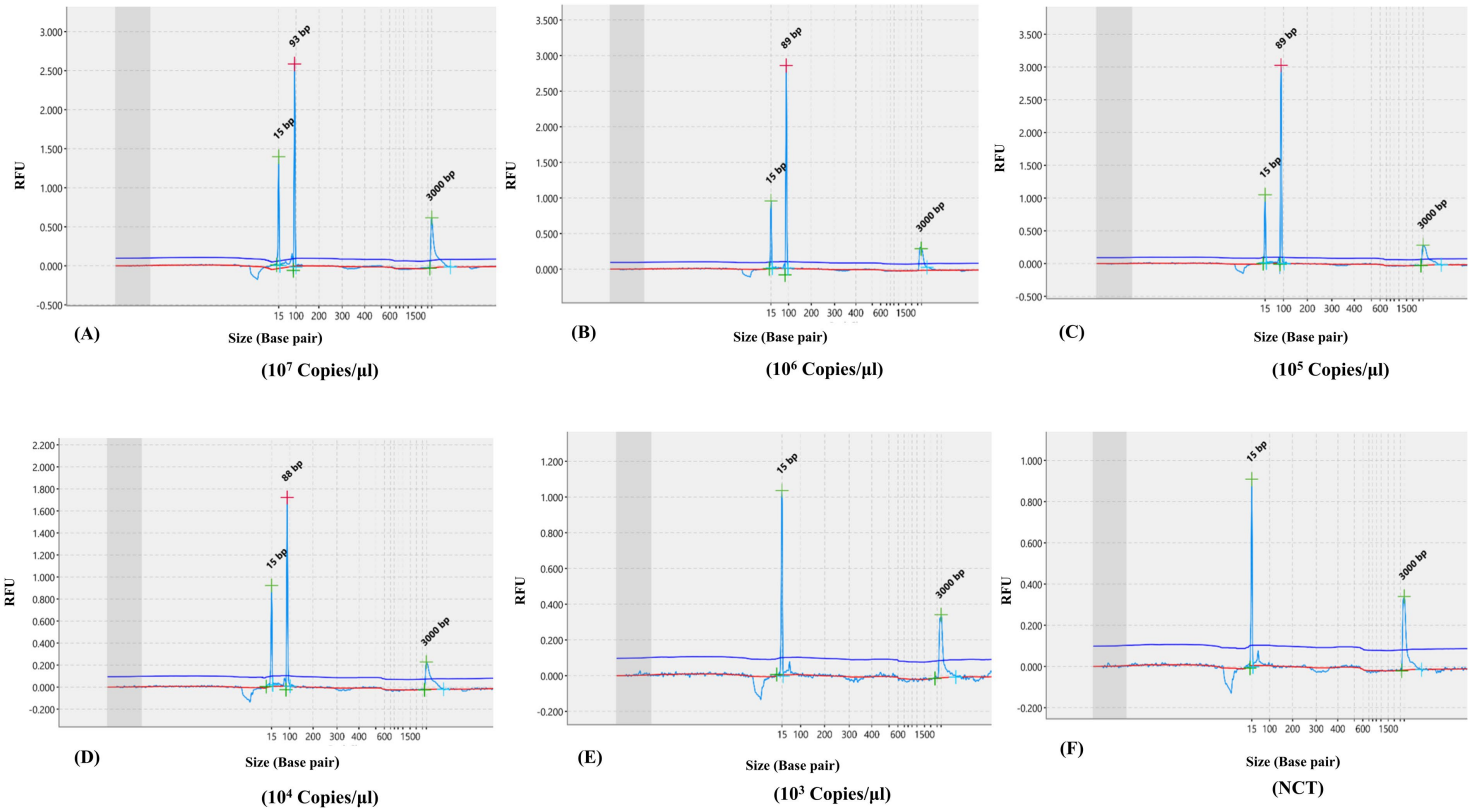
QIAxcel electropherogram analysis of serial plasmid DNA dilutions targeting the IOLA *16S rRNA* gene. QIAxcel electrophoresis was performed on serial ten-fold dilutions of plasmid DNA containing the IOLA *16S rRNA* target (10^7^ to 10^3^ copies/μl), along with a no-template control (NTC), to determine the assay’s limit of detection (LOD). The *x*-axis denotes DNA fragment size in base pairs (bp), calibrated using internal reference markers at 15 bp and 3000 bp, while the *y*-axis represents fluorescence intensity in relative fluorescence units (RFU). A detection threshold was set at >0.1 RFU. For all positive control dilutions showing amplification, a single, unique peak at approximately ~92 bp was detected, confirming specific amplification without nonspecific products. Amplification was observed down to 10^4^ copies/μL, establishing this as the assay’s LOD. No specific amplification was detected at 10^3^ copies/μL or in the NTC, confirming the assay’s specificity. The QIAxcel system demonstrated high resolution and sensitivity for quantitative detection of IOLA *16S rRNA gene* target. **(A)** 10^7^ copies/μl, **(B)** 10^6^ copies/μl, **(C)** 10^5^ copies/μl, **(D)** 10^4^ copies/μl, **(E)** 10^3^ copies/μl, **(F)** No-template control.

**Figure 2:**
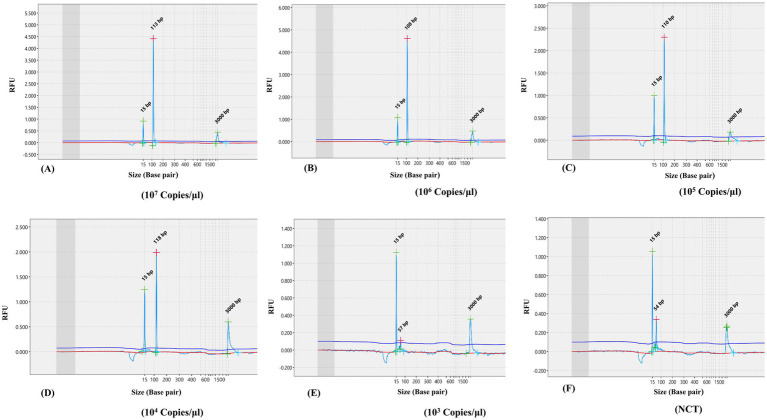
QIAxcel electropherogram analysis of serial plasmid DNA dilutions targeting the IOLA *PrfA* gene. QIAxcel electrophoresis was performed on serial ten-fold dilutions of plasmid DNA containing the *PrfA* gene target (10^7^ to 10^3^ copies/μl), along with a no-template control (NTC), to determine the assay’s limit of detection (LOD). The *x*-axis denotes DNA fragment size in base pairs (bp), calibrated using internal reference markers at 15 bp and 3000 bp, while the *y*-axis represents fluorescence intensity in relative fluorescence units (RFU). For all positive control dilutions showing amplification, a single, unique peak at approximately ~120 bp was detected, confirming specific amplification without nonspecific products. Amplification was observed down to 10^4^ copies/μL, establishing this as the assay’s LOD. No specific amplification was detected at 10^3^ copies/μL or in the NTC, confirming the assay’s specificity. The QIAxcel system demonstrated high resolution and sensitivity for quantitative detection of IOLA *PrfA* gene targets. **(A)** 10^7^ copies/μl, **(B)** 10^6^ copies/μl, **(C)** 10^5^ copies/μl, **(D)** 10^4^ copies/μl, **(E)** 10^3^ copies/μl, **(F)** No-template control.

**Figure 3:**
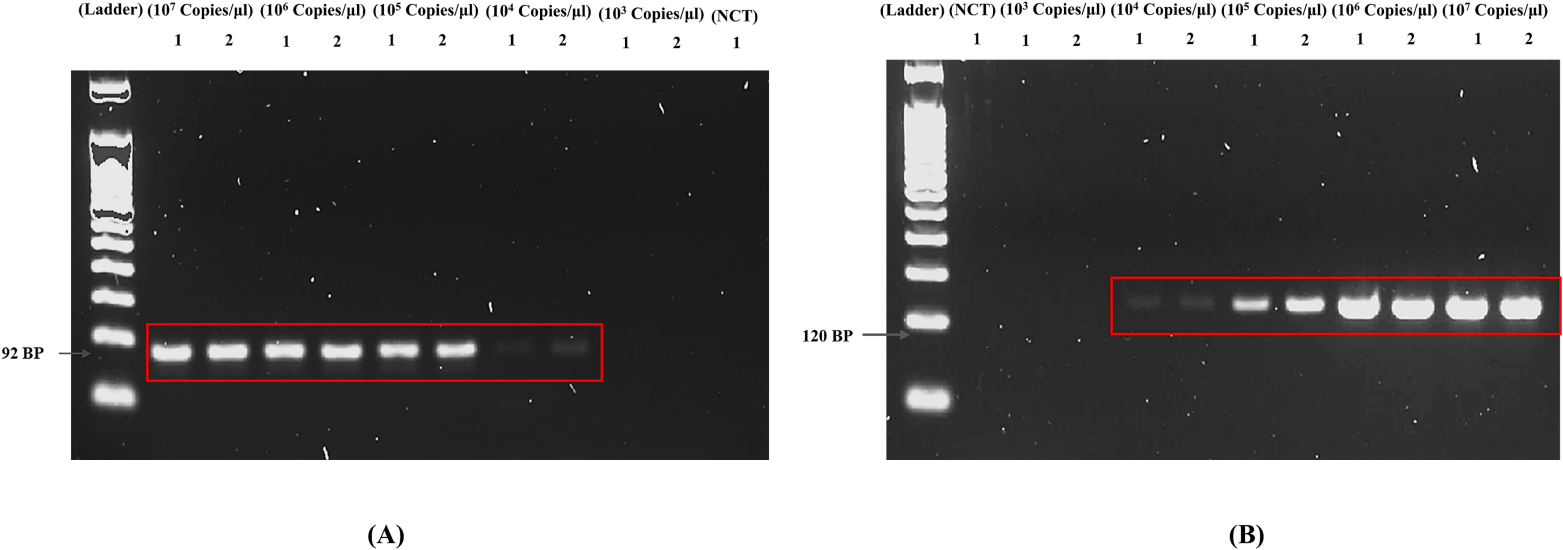
Gel electrophoresis analysis of IOLA *16S rRNA* and IOLA *PrfA* genes. Gel electrophoresis results for the amplification of the IOLA *16S rRNA* gene **(A)** and IOLA *PrfA* gene **(B)** using serial plasmid DNA dilutions ranging from 10^7^ to 10^3^ copies/μl. The assay successfully detected target amplification down to 10^4^ copies/μl, with each dilution performed in duplicate. Clear and specific bands were observed at the expected amplicon sizes for both genes, confirming successful amplification at the desired target sites.

Serial dilution of plasmid DNA established the limit of detection (LOD) at 10^4^ copies/μl for both the *16S rRNA* and *PrfA* gene assays (Figures 1, 2). QIAxcel analysis of positive controls generated amplicon bands at 88–91 bp (expected 92 bp) for the *16S rRNA* gene and 108–118 bp (expected 120 bp) for the *PrfA* gene. The slight variations in fragment size determination are consistent with known limitations of capillary electrophoresis systems. Short amplicons can display minor mobility shifts due to DNA secondary structure, differences in migration under normal versus denaturing conditions, instrument performance variability, and run-specific factors. Small deviations between expected and observed amplicon size are known to occur with QIAxcel-based fragment analysis because sizing is estimated relative to alignment markers and size standards (27). In this study, these deviations did not affect interpretation because all positive-control dilutions produced single, well-defined peaks close to the expected sizes, and these QIAxcel results matched the corresponding agarose gel electrophoresis bands. This demonstrates that the observed QIAxcel variation falls within an acceptable range for accurate amplicon identification. In addition, confirmatory agarose gel electrophoresis showed distinct bands at the expected sizes down to 10^4^ copies/μL for the IOLA *16S rRNA* and *PrfA* gene (Figure 3). At these dilutions, the QIAxcel result also exhibited detectable peaks above the 0.1 RFU threshold and confirmed that both amplicons met the assay’s limit-of-detection criteria.

### 3.3 PCR result of canine respiratory swabs

**Figure 4:**
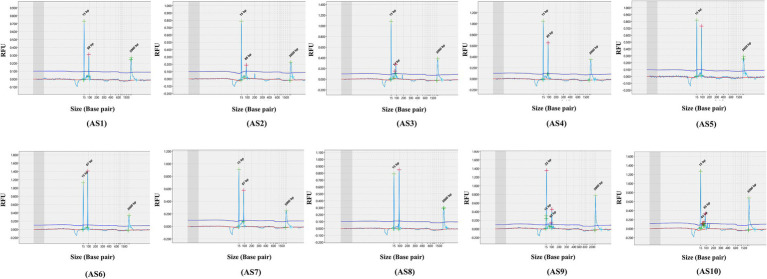
Representative electropherograms of ambiguous samples (AS) of IOLA *16S rRNA*, displaying amplification profiles for the *16S rRNA* gene target. The *x*-axis represents DNA fragment size in base pairs (bp), while the *y*-axis indicates fluorescence intensity measured in relative fluorescence units (RFU). Distinct amplicons are identified near the expected ~92 bp position (*16S rRNA* gene), with detection based on sharp peaks exceeding the threshold of 0.1 RFU. Internal size standards are marked at 15 bp and 3000 bp (indicated by green peaks). Unlike the positive control profiles, several samples exhibited multiple small clusters of peaks below the threshold level, which are unusual and may represent nonspecific products or background noise.

PCR analysis of the 55 Rickettsiales positive samples showed no amplification of IOLA *PrfA* while 45 of 55 samples showed no amplification of *16S rRNA* gene. This indicates that 81.8% of the samples were negative for IOLA by PCR-QIAxcel under the assay’s detection conditions. However, 10 samples showed amplicons at (8) or near (2) the expected size for the IOLA *16S rRNA* gene and were classified as ambiguous (Figure 4). Detailed characteristics of all samples including DNA concentration, presence, or absence of the expected amplicon peaks, RFU signal strength, and any additional non-specific peaks are provided in Supplementary Table 1. From the PCR end products corresponding to these 10 ambiguous samples (Table 2), four representative amplicons were randomly excised from the agarose gel representative bands (Supplementary Figure 6). DNA from these excised bands was purified using the QIAquick Gel Extraction Kit, cloned using the TOPO TA Cloning system, and subsequently subjected to Sanger sequencing. BLAST analysis of the bidirectional Sanger sequencing reads did not identify IOLA in any representative ambiguous amplicon. Representative sequences from the (8) near-size amplicon group showed only partial similarity to *Canis aureus* like sequences, whereas sequences from the (2) expected-size amplicon group showed no similarity to available NCBI database entries. Forward and reverse Sanger sequencing results for representative amplicons from both groups are provided (Supplementary Figure 7). Neither group matched IOLA *16S rRNA* or any IOLA-related sequence, therefore, these amplicons were interpreted as non-specific amplification, and all 10 ambiguous samples were classified as IOLA negative.

**Table 2:** Characteristics of 10 samples that showed IOLA *16S rRNA* gene like amplicon.

Sample	DNA Con. (ng/μl)	Breed	Sex	Specimen	State
1	0.18	Unknown Terrier Mix	Male	Nasal Swab	CO
2	0.86	Unknown Terrier Mix	Male	Throat Swab	CO
3	1.02	Mixed	Male	Pharyngeal Swab	WI
4	33.9	Border Collie Mix	Male	Pharyngeal Swab	PA
5	0.43	French Bulldog	Female	Nasopharyngeal Swab	KS
6	1.68	French Bulldog	Female	Nasopharyngeal Swab	KS
7	0.67	French Bulldog	Female	Nasopharyngeal Swab	KS
8	28.8	Mixed	Male	Sputum Swab	WI
9	4.81	American Pit Bull Terrier	Female	Nasopharyngeal Swab	WI
10	0.16	Labrador Retriever	Male	Pharyngeal Swab	OH

## 4 Discussion

Preliminary results from the University of New Hampshire suggested that a novel bacterium, IOLA, was a potential cause of the increased fatality rate associated with an atypical CIRD outbreak during the winter of 2023–2024 (17, 18). The current study used *16S* targeted sequencing and a newly developed dual target PCR assay to determine if IOLA was present in a large sample of suspected CIRD canine respiratory samples submitted to KSVDL during the outbreak period. Bacterial *16S* targeted sequencing and taxonomic classification of 777 samples identified 55 containing reads assigned to alphaproteobacterial members of the order Rickettsiales, within which IOLA has been proposed to reside. This reduced dataset was then subjected to PCR targeting both the IOLA *16S rRNA* and *PrfA* genes. None of the samples were PCR positive for both genes, indicating that IOLA was not detected in this subset of samples by our PCR assay. However, a few samples exhibited bands in the *16S* PCR reaction, but these were confirmed as non-specific amplification, including partial similarity to *Canis aureus* like DNA. Taken together, these findings do not support IOLA as a prevalent or molecularly detectable contributor to the 2023 aCIRD outbreak within the samples analyzed in this study. This conclusion aligns with the observations of Thieulent et al., who investigated the winter 2023 aCIRD outbreak by screening suspected cases for established CIRD pathogens using qPCR/RT-qPCR and subsequently applying viral metagenomic sequencing to selected samples (28). Their study concluded that the 2023 aCIRD outbreak was not likely caused by a new emerging pathogen, providing independent evidence consistent with our PCR-based findings.

Our study provides important methodological insights that shows the interpretation of earlier IOLA detection frameworks. The original identification of IOLA relied on nested *16S* PCR approaches that employed broad-range bacterial enrichment followed by organism-specific amplification (19). Our detailed primer mapping analysis reveals methodological considerations in this approach that merit discussion when evaluating IOLA detection strategies. The universal bacterial primers (E341f and E907r) employed in *Fukuda et al.,* first-round amplification show suboptimal complementarity to the IOLA *16S rRNA* gene sequence, with mismatches at positions 331–347 and 747–767 (19, 23). While this primer-template mismatch could reduce amplification efficiency for genuine IOLA sequences, the broad-range nature of these primers might simultaneously capture *16S rRNA* fragments from other bacterial taxa present in respiratory samples. Such non-target amplicons could potentially serve as templates in the subsequent IOLA-specific reaction, possibly contributing to apparent positive signals through low-level cross-reactivity. Notably, even our IOLA-specific *16S rRNA* primers, designed with perfect complementarity to the IOLA reference sequence and validated by primer-BLAST for specificity, occasionally produced amplification products of approximately expected size that were subsequently confirmed by sequencing to be non-IOLA bacterial sequences. This finding demonstrates that *16S rRNA* sequence similarity among respiratory bacteria can lead to cross-amplification despite careful primer design and suggests the existence of previously uncharacterized organisms with *16S* regions sufficiently similar to IOLA to permit partial amplification under standard PCR conditions. This observation underscores the value of our dual-target strategy, as none of the samples showing non-specific *16S rRNA* assay amplification were positive for the independent IOLA specific *PrfA* target, thereby providing orthogonal confirmation of true negative status. Regarding analytical sensitivity, our single-round conventional endpoint PCR assays achieved an LOD of approximately 10^4^ copies/μL for both targets. This threshold is less sensitive than nested PCR or qPCR approaches, including the framework used in the human IOLA study; because the human IOLA study reported organism burden as copies/mL (19), our LOD corresponds to approximately 10^7^ copies/mL for cross-study comparison. Thus, the assay was positioned to detect high-burden IOLA patterns comparable to clinically apparent human lower-respiratory cases, including pneumonia and bronchopulmonary infection (19), but could miss lower-copy detections reported in that dataset. Importantly, conventional endpoint PCR assays for canine respiratory pathogens have been used in CIRD investigations with reported detection limits in the 1,060–11,403 copies/mL range, as demonstrated by Kaul et al. (29). This supports the use of endpoint PCR as a reasonable preliminary molecular screening and confirmation approach. However, our assay should not be considered equivalent to optimized diagnostic qPCR panels for established pathogens such as *Mycoplasma cynos* or *Bordetella bronchiseptica*, which often achieve greater analytical sensitivity. Therefore, negative results in this study may reflect either true absence of IOLA or organism burden below the assay limit of detection. Within this context, the absence of dual-target-positive samples argues against prevalent high-burden, molecularly confirmed IOLA detection in this canine cohort, but does not exclude low-copy IOLA DNA. These observations highlight the need for sequence-level verification, multi-target confirmation, and future qPCR or digital PCR assays when investigating newly proposed pathogens in complex respiratory specimens.

The current study does have limitations: phylogenetic analysis based on *16S rRNA* gene sequences does not reliably resolve the taxonomic placement of IOLA. Its current assignment to the order Rickettsiales is instead supported by phylogenetic analyses of conserved ribosomal protein sequences. Beyond the order, IOLA does not follow any specific bacterial classification (19, 30). This casts doubt on the existence of the IOLA taxon. The uncertainty of the taxonomic placement of IOLA also affects the validity of our sampling strategy. We originally screened 777 samples by *16S* targeted sequencing to determine which samples should be evaluated by PCR. We chose to evaluate those samples that contained reads for bacteria in the order Rickettsiales, as this was the lowest reliable taxonomic level assigned for IOLA. *16S* targeted analysis has limitations when applied to novel organisms like IOLA that fall outside established bacterial taxonomy. Such limitations often hinder accurate species-level detection, necessitating reliance on higher taxonomic ranks and alternative molecular methods for comprehensive characterization (22). If the current taxonomic classification of IOLA within the order Rickettsiales is inaccurate, its presence in untested samples cannot be excluded based on phylogenetic assumptions alone. Additionally, even if present in the samples analyzed, IOLA may have been below the detection limit of the PCR assay, which could result in false-negative findings. Both taxonomic uncertainty and analytical sensitivity therefore represent important limitations when interpreting negative results. The observation that non-specific amplification occurred in some samples containing sequencing reads classified as Rickettsiales suggests that additional, currently unknown organisms with *16S* sequences similar to IOLA *16S rRNA* may exist in the canine respiratory tract or in the environment, further complicating taxonomic-based detection strategies. Both taxonomic uncertainty and analytical sensitivity therefore represent important limitations when interpreting negative results.

To reduce the risk of false-negative results, future investigators should consider more sensitive detection strategies, including qPCR or digital PCR assays targeting multiple genes, degenerate primer designs accommodating sequence diversity, and direct testing without taxonomic prescreening. More broadly, determining whether IOLA represents a cross-species pathogen, incidental colonizer, or sequence artifact will require integrated approaches spanning comparative genomics, shotgun metagenomic sequencing and refined pathogen discovery methods. A further priority is to clarify the role of established CIRD pathogens, particularly *Bordetella bronchiseptica*, through investigation of their strain-level diversity, evolutionary trajectories, and host-associated barriers across canine and human populations.

In conclusion, IOLA was not detected by dual-target PCR in canine respiratory samples from the 2023–2024 aCIRD outbreak, though this negative result requires cautious interpretation given methodological constraints including taxonomic uncertainty and limited analytical sensitivity. Importantly, our findings reveal that *16S*-based screening alone lacks sufficient specificity due to cross-amplification, highlighting critical barriers to reliable IOLA detection in respiratory specimens. We note that this investigation addressed only IOLA absence; demonstrating causality would require satisfying Koch’s postulates (31) or modern molecular criteria, which remains unfeasible without successful cultivation. Collectively, these findings provide a foundation for more rigorous pathogen-discovery strategies and underscore the importance of molecular validation in investigations of novel organisms within a One Health framework.

## 5 Conclusion

This study provides the first targeted molecular investigation of IOLA in canine respiratory samples collected during the 2023–2024 aCIRD outbreak. Despite screening a large and geographically diverse cohort, IOLA was not detected by dual-target PCR in samples selected through *16S* targeted prescreening. Together with the independent findings of Thieulent et al. (28) these data suggest that IOLA is unlikely to represent a prevalent organism of the 2023 aCIRD outbreak. More broadly, the study establishes a molecular foundation for future work and underscores the need for more sensitive, sequence-inclusive, and classification-independent approaches to determine whether IOLA or related organisms contribute to canine respiratory disease.

## Data Availability

The raw sequence reads generated for this study have been deposited in the NCBI Sequence Read Archive (SRA) under BioProject accession number PRJNA1481140 (https://www.ncbi.nlm.nih.gov/sra/PRJNA1481140). The search strategies and screening tags used for the statistical analysis are provided in the Supplementary materials. Certain secondary datasets related to bacterial community analysis are not publicly available due to institutional restrictions (Kansas Veterinary Diagnostic Laboratory; KVDL). These data are available from the corresponding author upon reasonable request and subject to institutional data-sharing agreements.
