# Supplementary material for: A targeted PCR approach for the detection of IOLA in canine infectious respiratory disease samples during an atypical CIRD outbreak in winter 2023
Source: Front Vet Sci. 2026 Jun 26;13:1849862. doi: 10.3389/fvets.2026.1849862 (PMC13354092; doi:10.3389/fvets.2026.1849862)
Supplement: Supplementary file 1 [file Table_1.docx]

**Supplementary Table 1.** Detection of IOLA 16S rRNA and PrfA Genes in Canine respiratory samples with corresponding DNA concentrations and additional peak profiles.

| **Sample**  **Number** | **Initial DNA**  **Con. (ng/** **µl)** | **IOLA *16s rRNA* gene** | | **IOLA *PrfA* gene** | |
| --- | --- | --- | --- | --- | --- |
|  |  | **(92 BP Peak)** | Extra peaks (size range) | **(120 BP Peak)** | Extra peaks  (size range) |
|  |  | Distinguished and  (strong RFU signal) |  | Distinguished and  (strong RFU signal) |  |
| 1 | 0.24 | Absent | (35-75) BP | Absent | Absent |
| 2 | 2.54 | Absent | (35-75) BP | Absent | 55 BP |
| 3 | 6.47 | Absent | (35-75) BP | Absent | 55 BP |
| 4 | 0.18 | **Present** | **Absent** | Absent | Absent |
| 5 | 0.86 | **Present** | **Absent** | Absent | Absent |
| 6 | 0.17 | Absent | (50-75) BP | Absent | Absent |
| 7 | 1.3 | Absent | (50-75) BP | Absent | 61 BP |
| 8 | 1.02 | **Present** | (50-75) BP | Absent | 53 BP |
| 9 | 3.97 | Absent | (22; 35-75; 101) BP | Absent | 55 BP |
| 10 | 0.42 | Absent | (50-75) BP | Absent | 41; 54 BP |
| 11 | 2.16 | Absent | (35-75) BP | Absent | 56 BP |
| 12 | 33.90 | **Present** | **Absent** | Absent | 55 BP |
| 13 | 0.22 | Absent | (35-75; 214) BP | Absent | 57 BP |
| 14 | 0.43 | **Present** | **Absent** | Absent | 53 BP |
| 15 | 1.68 | **Present** | **Absent** | Absent | 56 BP |
| 16 | 0.67 | **Present** | **Absent** | Absent | 56 |
| 17 | 1.15 | Absent | Absent | Absent | 56 |
| 18 | 0.35 | Absent | (35-50) BP | Absent | 58 |
| 19 | 0.27 | Absent | (50-75) BP | Absent | Absent |
| 20 | 0.18 | Absent | (50-75) BP | Absent | Absent |
| 21 | 0.40 | Absent | (35-75; 111) BP | Absent | Absent |
| 22 | 0.08 | Absent | (20-49) BP | Absent | 52 BP |
| 23 | 1.77 | Absent | (35-75) BP | Absent | Absent |
| 24 | 1.22 | Absent | (35-75; 82 BP; 164) BP | Absent | 56 BP |
| 25 | 0.15 | Absent | (35-75; 104) BP | Absent | 57 BP |
| 26 | 0.09 | Absent | (35-75; 128) BP | Absent | 56 BP |
| 27 | 0.45 | Absent | (35-75) BP | Absent | 55 BP |
| 28 | 2.49 | Absent | (35-75; 108) BP | Absent | 54 BP |
| 29 | 0.11 | Absent | (35-75; 114) BP | Absent | 55 BP |
| 30 | 0.71 | Absent | (50-75) BP | Absent | Absent |
| 31 | 28.8 | **Present** | **Absent** | Absent | 58 BP |
| 32 | 2.01 | Absent | (22; 35-75) BP | Absent | 56 BP |
| 33 | 46.8 | Absent | (22; 35-75) BP | Absent | 56 BP |
| 34 | 56.9 | Absent | (22; 35-75) BP | Absent | 55 BP |
| 35 | 0.25 | Absent | (22; 35-75) BP | Absent | 55 BP |
| 36 | 0.19 | Absent | (22; 35-75; 113) BP | Absent | 55 BP |
| 37 | 0.29 | Absent | (22; 35-75; 157) BP | Absent | 56 BP |
| 38 | 0.22 | Absent | (22; 35-75) BP | Absent | 56 BP |
| 39 | 0.46 | Absent | (22; 35-75; 114) BP | Absent | 56 BP |
| 40 | 0.49 | Absent | (22; 35-75) BP | Absent | 55 BP |
| 41 | 0.02 | Absent | (22; 35-75; 114) BP | Absent | 55 BP |
| 42 | 0.17 | Absent | (22; 35-75; 119) BP | Absent | 56 BP |
| 43 | 4.81 | **Present** | (22; 35-75) BP | Absent | 57 BP |
| 44 | 0.17 | Absent | (22; 35-75) BP | Absent | 56 BP |
| 45 | 1.03 | Absent | (22; 35-75) BP | Absent | 56 BP |
| 46 | 0.16 | **Present** | (22; 35-75) BP | Absent | 54 BP |
| 47 | 0.40 | Absent | (22; 35-75) BP | Absent | 56 BP |
| 48 | 0.22 | Absent | (22; 35-75) BP | Absent | 54 BP |
| 49 | 0.45 | Absent | (22; 35-75) BP | Absent | 54 BP |
| 50 | 0.26 | Absent | (22; 35-75) BP | Absent | 55 BP |
| 51 | 0.96 | Absent | (22; 35-75; 118) BP | Absent | 55 BP |
| 52 | 0.15 | Absent | (22; 35-75; 163) BP | Absent | 43; 56 BP |
| 53 | 0.34 | Absent | (22; 35-75) BP | Absent | 56 BP |
| 54 | 0.36 | Absent | (22; 35-75) BP | Absent | 56 BP |
| 55 | 0.10 | Absent | (22; 35-75) BP | Absent | 52 BP |
