# Supplementary material for: A targeted PCR approach for the detection of IOLA in canine infectious respiratory disease samples during an atypical CIRD outbreak in winter 2023
Source: Front Vet Sci. 2026 Jun 26;13:1849862. doi: 10.3389/fvets.2026.1849862 (PMC13354092; doi:10.3389/fvets.2026.1849862)
Supplement: Supplementary file 2 [file Data_sheet_1.docx]

Supplementary Material

# Supplementary Data

**Positive control sequence for IOLA *16S* *rRNA***

TTGTAGATACTAGAAGGAACACCAGGGGCGAAGGCGATTAACTATTTTAATCTGACACTGAGACACGAAAGCGTGGGGAGTAAAAAGGATTAGAGACCCTTGTAATCCACGCTGTAAAAGATGAGTATTTGATATTGTTAAA

**Positive control sequence for IOLA *PrfA***

AACGCTATTTTTTTATTTTGTATTTGATTTCTTTCTTGCTGTGATACTATATTTACATCTTTTAATATTTTGTGTTTTAATCTAACAGCGCTTGAAGTCTTGTTAACATGCTGTCCTCCTGCTCCTGATGCTTTAAATGTTTCCCAAATAAAAAATTTATCTTGCAATAA

Red text nucleotides represent the forward and reverse primer sequences.

# Supplementary Figures and Tables

## Supplementary Figures

Forward read


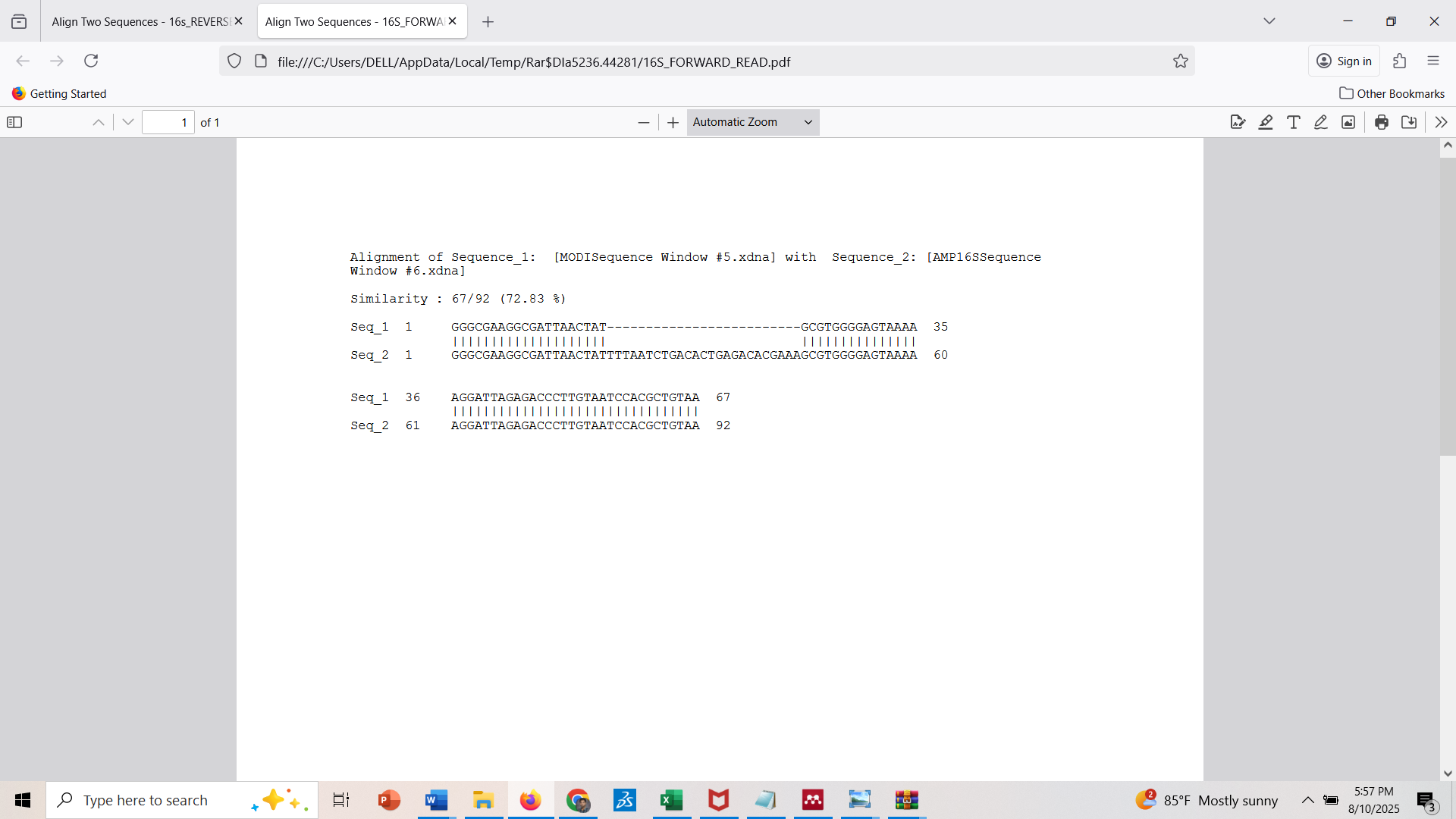


Reverse read


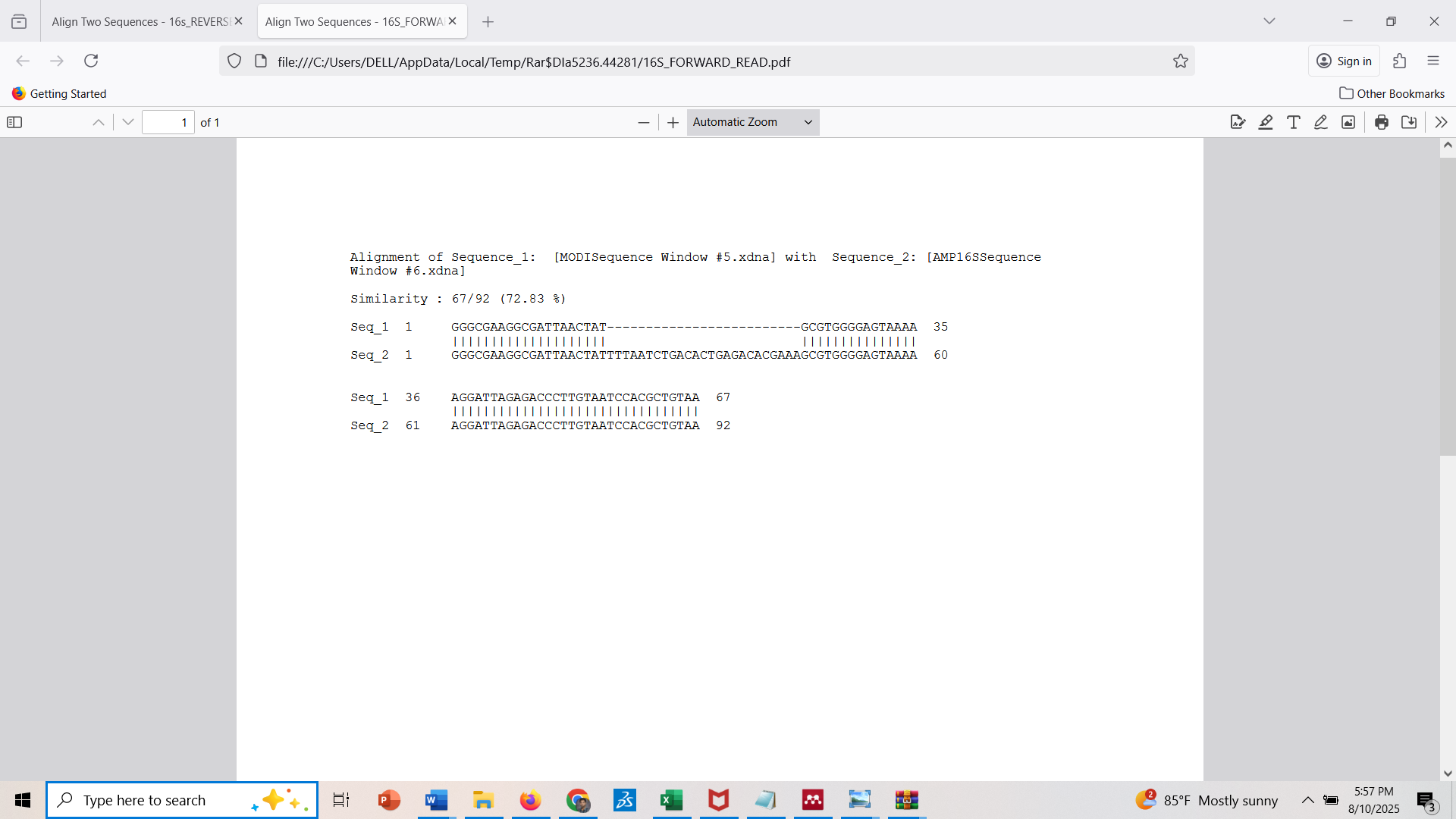


**Supplementary Figure 1.** IOLA *16S* gene PCR product sequencing (Result shows complete match of 92 BP).


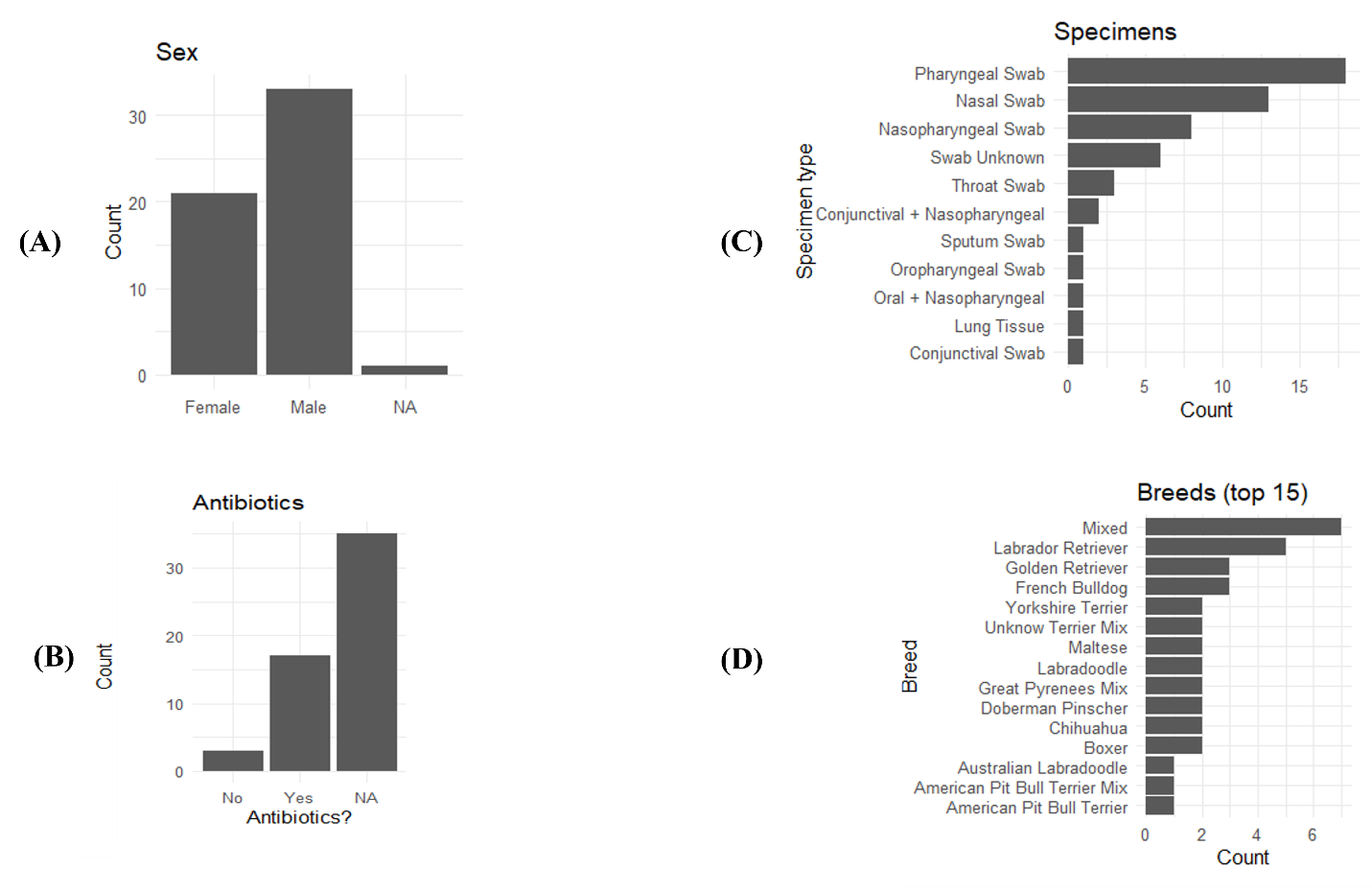


**Supplementary Figure 2.** Demographic and sampling characteristics of the Rickettsiales order detected sample (n = 55). Bar plots summarize (A) sex, (B) prior antibiotic exposure, (C) specimen type, and (D) breed (top 15 reported). Most samples were collected as pharyngeal and nasal swabs, with smaller numbers of nasopharyngeal, throat, oropharyngeal, conjunctival‑combined swabs, sputum swabs, oral‑combined swabs, lung tissue, and conjunctival swabs; a subset was recorded as “swab unknown.” Males were more frequent than females. Antibiotic history was largely missing (NA); among records with data, prior antibiotics were reported more often than not. Mixed-breed dogs were the most common, followed by Labrador Retriever, Golden Retriever, French Bulldog, Yorkshire Terrier, and other breeds shown. Counts are displayed as raw numbers; NA denotes missing information.


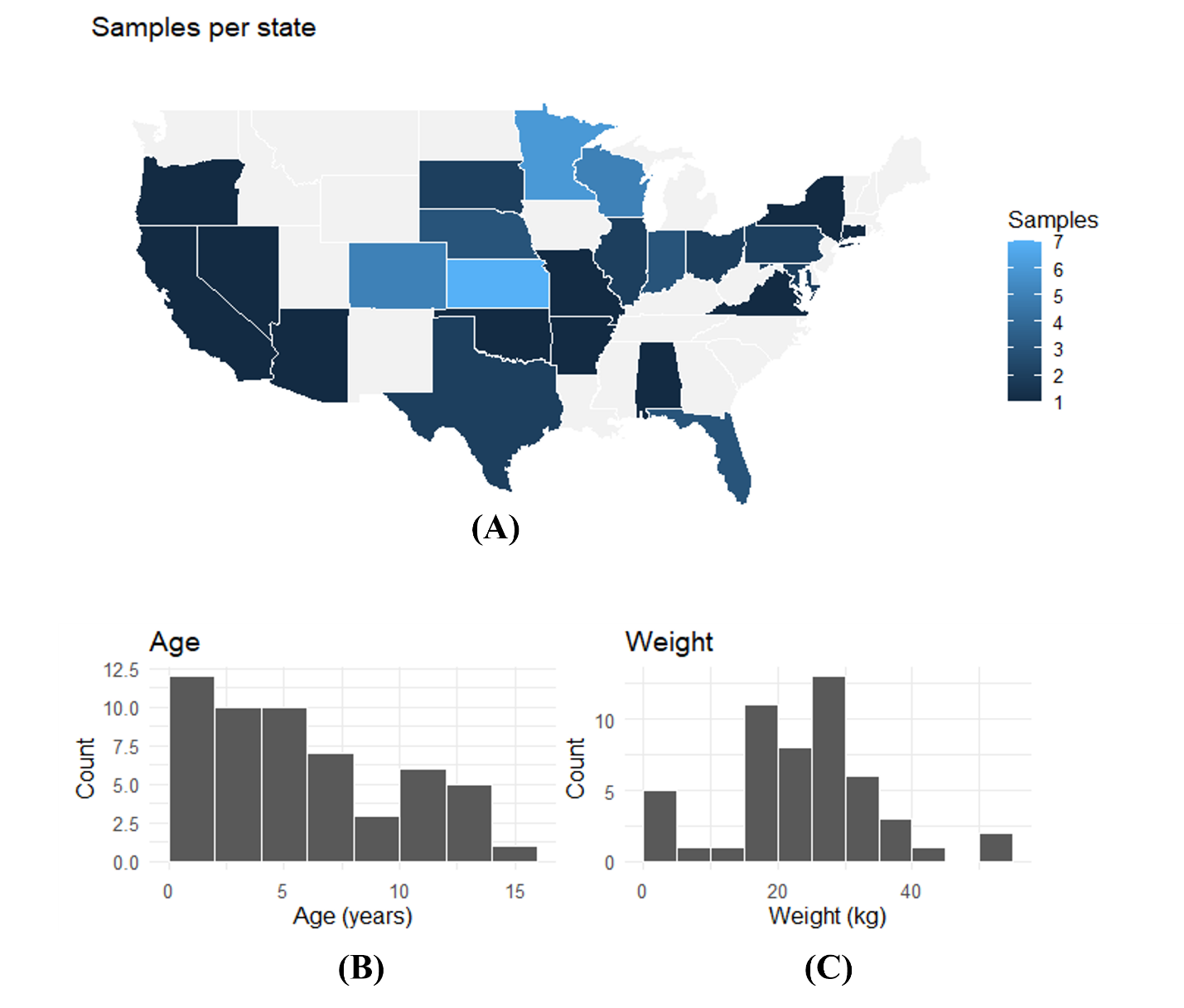


**Supplementary Figure 3.** Geographic and demographic characteristics of the 55 samples containing members of the Rickettsiales order detected by NGS analysis. (A) Choropleth map of the United States showing the distribution of the 55 samples by state, with sample counts ranging from 1 to 7. States with no samples are shaded in light gray. (B) Age distribution of sampled dogs, ranging from <1 year to 15 years, with the largest representation between 0–5 years. (C) Weight distribution of sampled dogs, ranging from <5 kg to >40 kg, with most individuals between 15–30 kg. Counts are shown as raw numbers, and the figure represents demographic data only.


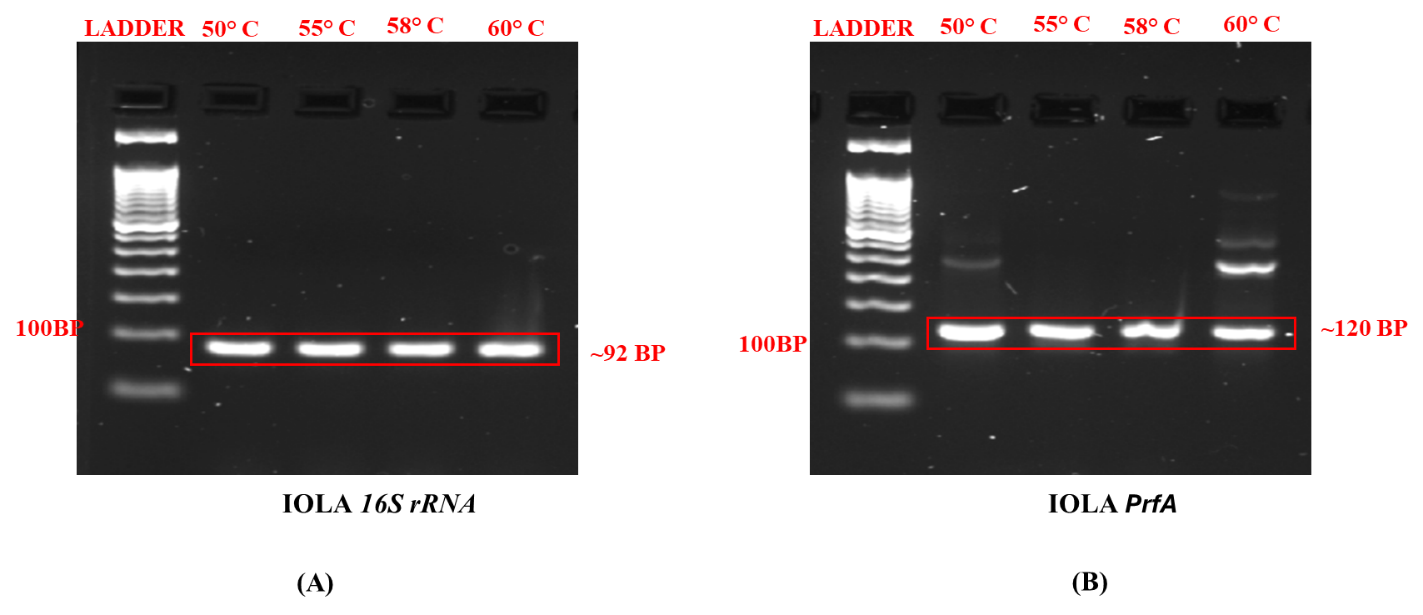


**Supplementary Figure 4.** Optimization of annealing temperatures for PCR amplification of IOLA target genes (16S *rRNA* and *PrfA*). Agarose gel electrophoresis (3%) showing PCR products generated from IOLA-positive control DNA using primer sets targeting the (A) IOLA 16S *rRNA* gene and (B) IOLA *PrfA* gene at four different annealing temperatures (50 °C, 55 °C, 58 °C, and 60 °C). Lanes labeled "LADDER" contain a 100 bp DNA marker. Each primer set consistently amplified the expected 92 BP and 120 BP amplicon for IOLA 16S gene and IOLA *PrfA* across the tested temperature range; however, the 58 °C annealing temperature yielded the most distinct and specific bands with minimal background amplification for both genes. Based on these results, 58 °C was selected as the optimal annealing temperature for all subsequent PCR assays targeting IOLA 16S *rRNA* and *PrfA*.


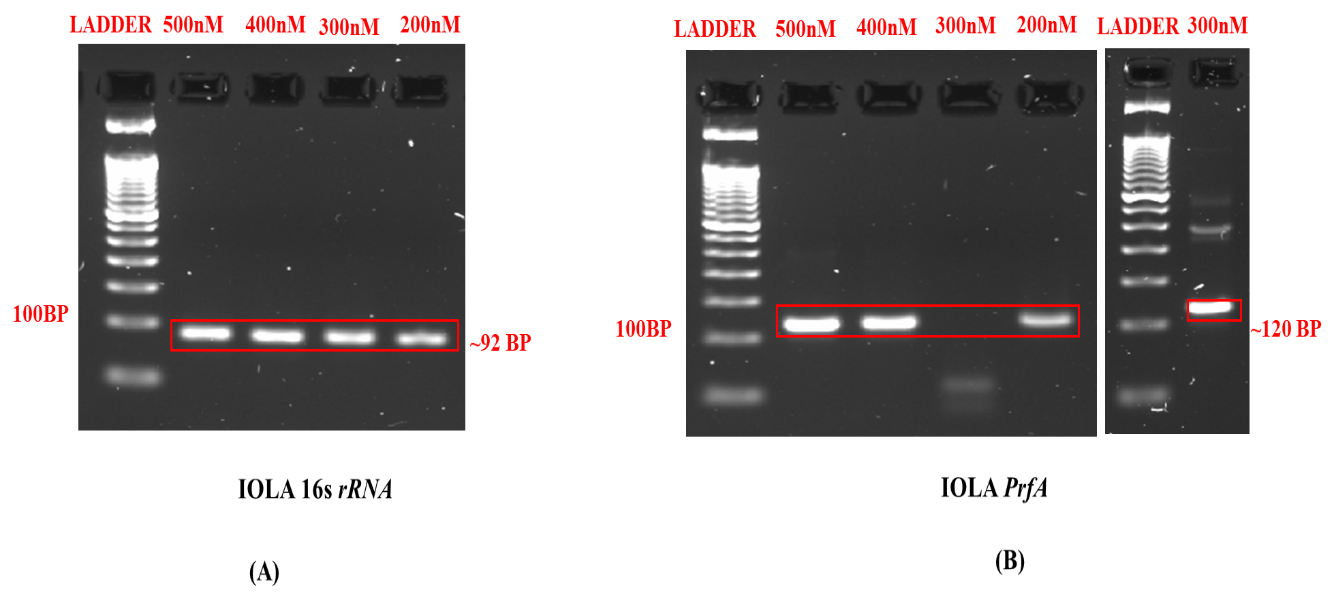


Supplementary Figure 5. Optimization of primer concentration for PCR amplification of IOLA target genes (*16S rRNA* and *PrfA*). Agarose gel electrophoresis (3%) showing PCR products generated from IOLA-positive control DNA using primer sets targeting the (A) IOLA *16S rRNA* gene (~92 bp) and (B) IOLA *PrfA* gene (~120 bp) at four different primer concentrations (500 nM, 400 nM, 300 nM, and 200 nM). Lanes labeled "LADDER" contain a 100 bp DNA marker, with the 100 bp band indicated for reference. For both target genes, the 500 nM primer concentration produced the most intense and distinct amplicon bands with minimal nonspecific amplification, while lower concentrations yielded progressively weaker signals. Based on these results, a final primer concentration of 500 nM was selected for all subsequent PCR assays targeting IOLA *16S rRNA* and *PrfA*.


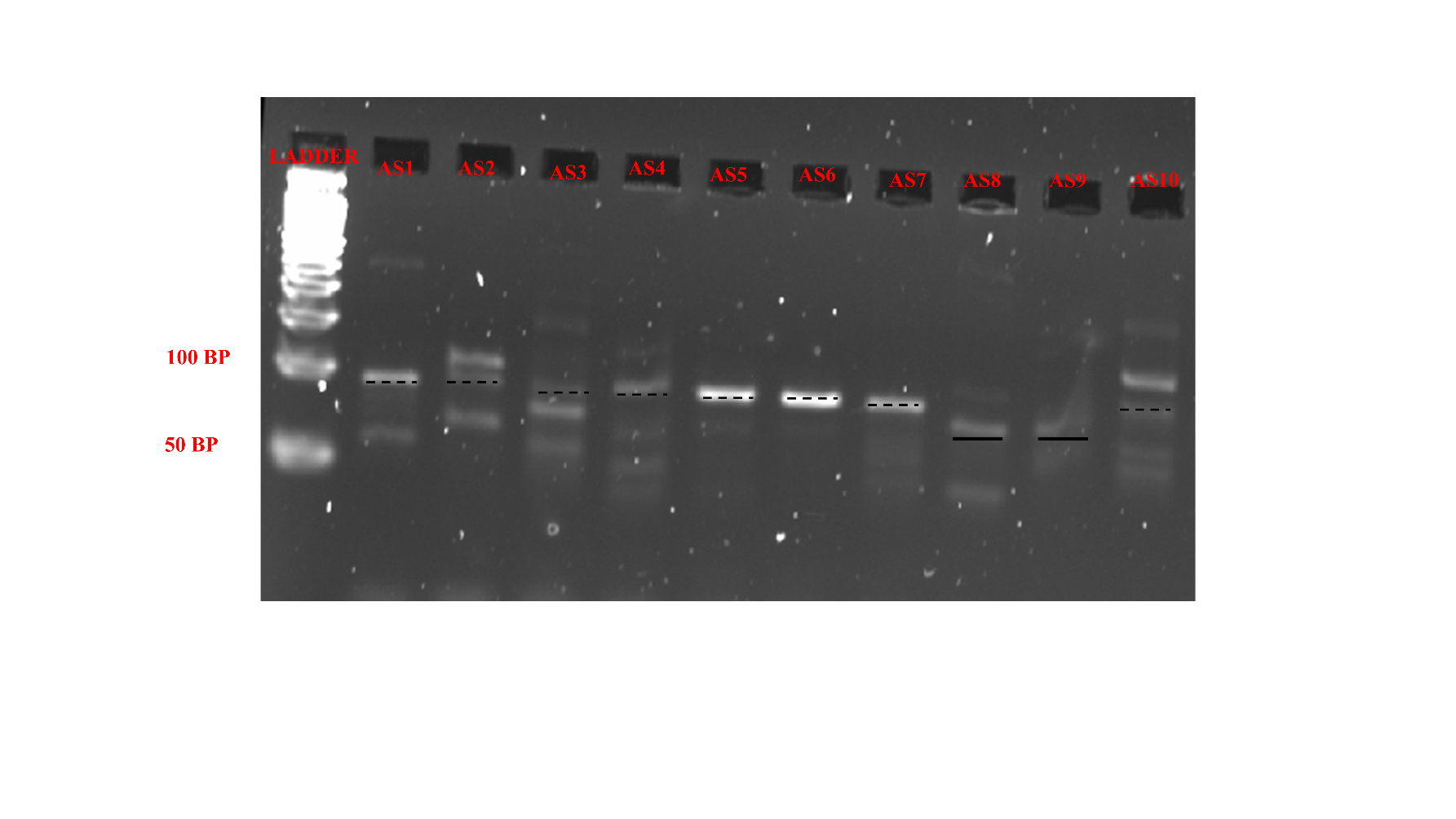


Supplementary Figure 6. PCR amplification of the IOLA *16S rRNA* gene in 10 clinical samples (AS1–AS10) showing distinct and variant banding patterns. Agarose gel electrophoresis (3%) of PCR products amplified using IOLA *16S rRNA* specific primers (~92 bp expected size) from 10 clinical specimens (AS1–AS10). Lanes labeled "LADDER" contain a 100 bp DNA marker, with 50 bp and 100 bp bands indicated for reference. Most samples with dotted black line displayed a band aligned with the closely ~92 bp position, whereas solid black line (AS8 and AS9) exhibited bands migrating slightly below the first group, along with additional nonspecific bands. Based on these electrophoretic patterns, samples were classified into two groups: (1) those with group 1 (AS1–AS7 and SS10) and group 2 (AS8 and AS9). Target bands from both groups (two samples from each) were exercised and purified for downstream analysis to confirm sequence identity.

**(i) Group 1 (AS1–AS7 and SS10)**

**>IOLA_16S_Forward read**

NNNNNNNNNNCNNNACTAAAGGGACTAGTCCTGCAGGTTTAAACGAATTCGCCCTTGGGCGAAGGCGATTAACTATCTGT

CCCTGTGTCCCTATGTCCACATGTCCATGTTTCCCTGTGTCCCTTGTAATCCACGCTGTAA AAGGGCGAATTCGCGGCCG

CTAAATTCAATTCGCCCTATAGTGAGTCGTATTACAATTCACTGGCCGTCGTTTTACAACGTCGTGACTGGGAAAACCCT

GGCGTTACCCAACTTAATCGCCTTGCAGCACATCCCCCTTTCGCCAGCTGGCGTAATAGCGAAGAGGCCCGCACCGATCG

CCCTTCCCAACAGTTGCGCAGCCTATACGTACGGCAGTTTAAGGTTTACACCTATAAAAGAGAGAGCCGTTATCGTCTGT

TTGTGGATGTACAGAGTGATATTATTGACACGCCGGGGCGACGGATGGTGATCCCCCTGGCCAGTGCACGTCTGCTGTCA

GATAAAGTCTCCCGTGAACTTTACCCGGTGGTGCATATCGGGGATGAAAGCTGGCGCATGATGACCACCGATATGGCCAG

TGTGCCGGTCTCCGTTATCGGGGAAGAAGTGGCTGATCTCAGCCACCGCGAAAATGACATCAAAAACGCCATTAACCTGA

TGTTCTGGGGAATATAAATGTCAGGCATGAGATTATCAAAAAGGATCTTCACCTAGATCCTTTTCACGTAGAAAGCCAGT

CCGCAGAAACGGTGCTGACCCCGGATGAATGTCAGCTACTGGGCTATCTGGACAAGGGAAAACGCAAGCGCAAAGAGAAA

GCAGGTAGCTTGCAGTGGGCTTACATGGCGATAGCTAGACTGGGCGGTTTTATGGACAGCAAGCGAACCGGAATTGCCAG

CTGGGGCGCCCTCTGGNAAGGTTGGGAAGCCCTGCAAAGTAAACTGGNTGGCTTTCTTGCCGCCAAGGATCTGATGGCGC

NGGGGATCAAGCTCTGATCAAGAGACAGGATGANGATCGTTTCGCATGATTGAACAAGATGGNTTGCACGCAGGTTCNNC

NGNCCGCTTGGGGNNGNANAGGCTATTCNGCTATGACTGGGNNNAACNNNANNNATCGGCTGNTCTGATGCNNNCGNGTT

NCNNNNNCANNGCNGGGNCGCCCNNNNNNTTTTTNNNCANGACNACNNNNNNNCCNNANGNACNNCANANNNNNNGNNCN

NNNNTCNNNNNNNNNNNNNNNNNNNNGGNNNNNNCNTTNNNNCNCNNNN

**NCBI blast result:**


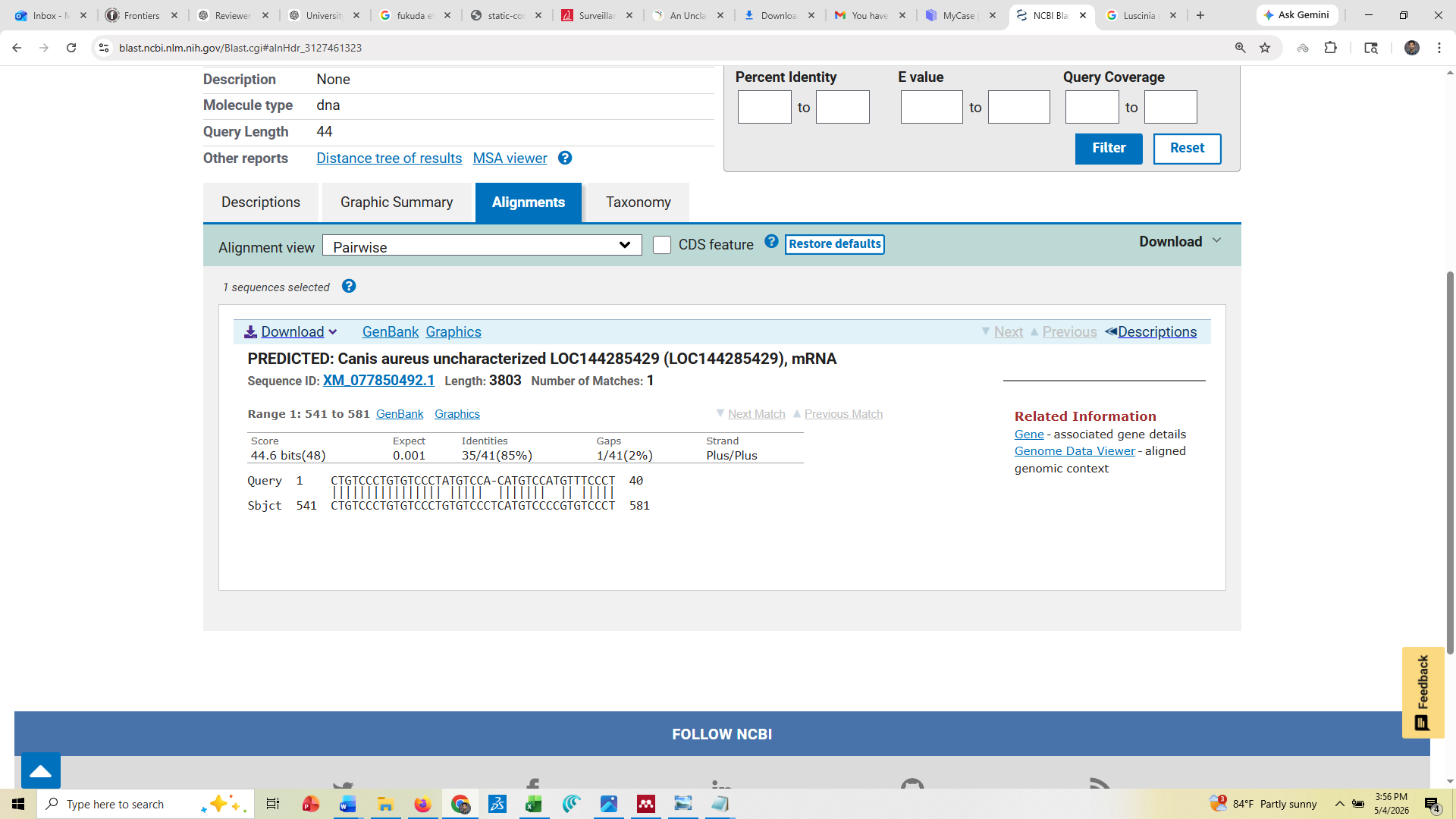


**>IOLA_16S_ Reverse read**

NNNNNNNGGGNGATTGATTTAGCGGCCGCGAATTCGCCCTTTTACAGCGTGGATTACAAGGGACACAGGGAAACATGGAC

ATGTGGACATAGGGACACAGGGACAGATAGTTAATCGCCTTCGCCCAAGGGCGAATTCGTTTAAACCTGCAGGACTAGTC

CCTTTAGTGAGGGTTAATTCTGAGCTTGGCGTAATCATGGTCATAGCTGTTTCCTGTGTGAAATTGTTATCCGCTCACAA

TTCCACACAACATACGAGCCGGAAGCATAAAGTGTAAAGCCTGGGGTGCCTAATGAGTGAGCTAACTCACATTAATTGCG

TTGCGCTCACTGCCCGCTTTCCAGTCGGGAAACCTGTCGTGCCAGCTGCATTAATGAATCGGCCAACGCGCGGGGAGAGG

CGGTTTGCGTATTGGGCGCTCTTCCGCTTCCTCGCTCACTGACTCGCTGCGCTCGGTCGTTCGGCTGCGGCGAGCGGTAT

CAGCTCACTCAAAGGCGGTAATACGGTTATCCACAGAATCAGGGGATAACGCAGGAAAGAACATGTGAGCAAAAGGCCAG

CAAAAGGCCAGGAACCGTAAAAAGGCCGCGTTGCTGGCGTTTTTCCATAGGCTCCGCCCCCCTGACGAGCATCACAAAAA

TCGACGCTCAAGTCAGAGGTGGCGAAACCCGACAGGACTATAAAGATACCAGGCGTTTCCCCCTGGAAGCTCCCTCGTGC

GCTCTCCTGTTCCGACCCTGCCGCTTACCGGATACCTGTCCGCCTTTCTCCCTTCGGGAAGCGTGGCGCTTTCTCATAGC

TCACGCTGTAGGTATCTCAGTTCGGTGTAGGTCGTTCGCTCCAAGCTGGGCTGTGTGCACGAACCCCCCGTTCAGCCCGA

CCGCTGCGCCTTATCCGGTAACTATCGTCTTGAGTCCAACCCGGTAAGACACGACTTATCGCCACTGGCAGCAGCCACTG

GNANCAGGATTAGCAGANCGAGGTATGTAGGNGNNGCTACAGANTNNGAGTGNGNNTANTACGGCTACNCTANANANAGT

ANTTGNATCTGCGCTCTGCTGAGCNNTACNNNNAAANNANNNNAGCTNNGATNNNNANNNNNCGNTGNAGCGGNNNGNTT

TTTNGNTGCAAGCNNCNATACNCNNNNAANNNATNNNNNAANANNNNNGNNNNTTNNNNNNNNANCNNNNNNNNNNANNN

NNNCNNTTAGGGNANTTNGNNNNNNNNNNNNANNNA

**NCBI blast result:**


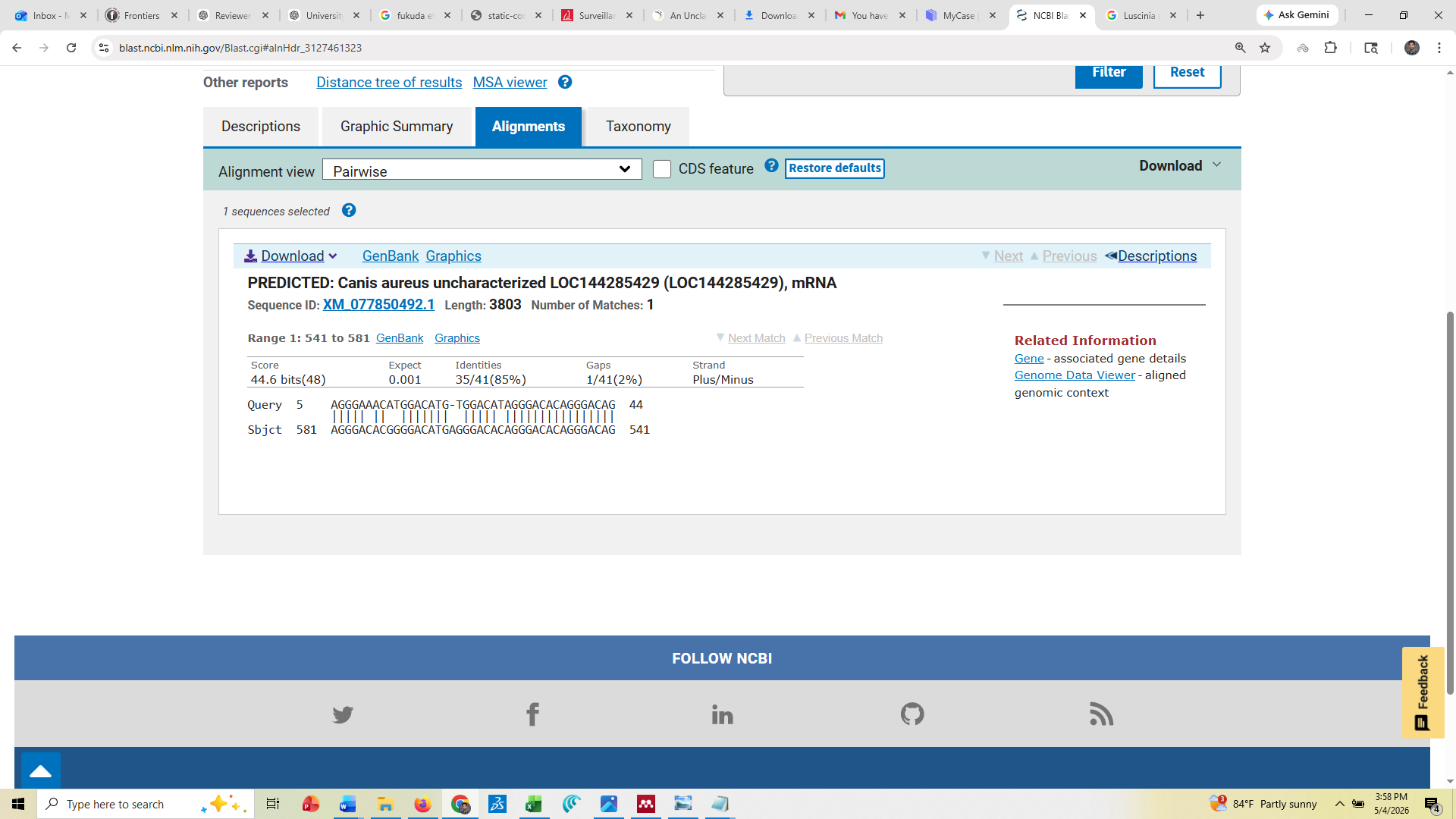


**ii) Group 2 (AS8 and AS9).**

**>IOLA_16S_Forward read**

NNNNNNNNNNGGNGATTGNNTTAGCGGCCGCGAATTCGCCCTTGGGCGAAGGCGATTAACTATCTACTTGGGATGCGATG

ACGGATAAGAGCTCTAAGCGCCCCTTGTAATCCACGCTGTAAAAGGGCGAATTCGTTTAAACCTGCAGGACTAGTCCCTT

TAGTGAGGGTTAATTCTGAGCTTGGCGTAATCATGGTCATAGCTGTTTCCTGTGTGAAATTGTTATCCGCTCACAATTCC

ACACAACATACGAGCCGGAAGCATAAAGTGTAAAGCCTGGGGTGCCTAATGAGTGAGCTAACTCACATTAATTGCGTTGC

GCTCACTGCCCGCTTTCCAGTCGGGAAACCTGTCGTGCCAGCTGCATTAATGAATCGGCCAACGCGCGGGGAGAGGCGGT

TTGCGTATTGGGCGCTCTTCCGCTTCCTCGCTCACTGACTCGCTGCGCTCGGTCGTTCGGCTGCGGCGAGCGGTATCAGC

TCACTCAAAGGCGGTAATACGGTTATCCACAGAATCAGGGGATAACGCAGGAAAGAACATGTGAGCAAAAGGCCAGCAAA

AGGCCAGGAACCGTAAAAAGGCCGCGTTGCTGGCGTTTTTCCATAGGCTCCGCCCCCCTGACGAGCATCACAAAAATCGA

CGCTCAAGTCAGAGGTGGCGAAACCCGACAGGACTATAAAGATACCAGGCGTTTCCCCCTGGAAGCTCCCTCGTGCGCTC

TCCTGTTCCGACCCTGCCGCTTACCGGATACCTGTCCGCCTTTCTCCCTTCGGGAAGCGTGGCGCTTTCTCATAGCTCAC

GCTGTAGGTATCTCAGTTCGGTGTAGGTCGTTCGCTCCAAGCTGGGCTGTGTGCACGAACCCCCCGTTCAGCCCGACCGC

TGCGCCTTATCCGGTAACTATCGTCTTGAGTCCAACCCGGTAAGACACGACTTATCGCCACTGGNAGCAGCCACTGGNNA

CAGGATTAGCAGAGCGAGGTATGTNGGCGGNGCTACAGANTTCTTGAAGTNNNGNNTAACTACGGCTACACTANAGANAG

TATTTGGNATCTGCGCTCTGCTNANCCAGTTANCTTCNGANNAGNNTNNAGCTCTGATCNNANNAACNNNCNNCTNNNGC

GNNNTTTTTTTNNNTNGCANCAGNNNNATACNNNCANAAAANNNNNNNNNNNNNNNNNCNNNNNNNNTNCNACGGGNNNN

NNANNNNNNNCNNNNANNNNNN

**NCBI blast result:** No similarity

**>IOLA_16S_ Reverse read**

NNNNNNANCCTNNCTAAAGGGACTAGTCCTGCAGGTTTAAACGAATTCGCCCTTTTACAGCGTGGATTACAAGGG GCGCTTAGAGCTCTTATCCGTCATCGCATCCCAAGTAGATAGTTAATCGCCTTCGCCCAAGGGCGAATTCGCGGCCGCTAAATTC

AATTCGCCCTATAGTGAGTCGTATTACAATTCACTGGCCGTCGTTTTACAACGTCGTGACTGGGAAAACCCTGGCGTTAC

CCAACTTAATCGCCTTGCAGCACATCCCCCTTTCGCCAGCTGGCGTAATAGCGAAGAGGCCCGCACCGATCGCCCTTCCC

AACAGTTGCGCAGCCTATACGTACGGCAGTTTAAGGTTTACACCTATAAAAGAGAGAGCCGTTATCGTCTGTTTGTGGAT

GTACAGAGTGATATTATTGACACGCCGGGGCGACGGATGGTGATCCCCCTGGCCAGTGCACGTCTGCTGTCAGATAAAGT

CTCCCGTGAACTTTACCCGGTGGTGCATATCGGGGATGAAAGCTGGCGCATGATGACCACCGATATGGCCAGTGTGCCGG

TCTCCGTTATCGGGGAAGAAGTGGCTGATCTCAGCCACCGCGAAAATGACATCAAAAACGCCATTAACCTGATGTTCTGG

GGAATATAAATGTCAGGCATGAGATTATCAAAAAGGATCTTCACCTAGATCCTTTTCACGTAGAAAGCCAGTCCGCAGAA

ACGGTGCTGACCCCGGATGAATGTCAGCTACTGGGCTATCTGGACAAGGGAAAACGCAAGCGCAAAGAGAAAGCAGGTAG

CTTGCAGTGGGCTTACATGGCGATAGCTAGACTGGGCGGTTTTATGGACAGCAAGCGAACCGGAATTGCCAGCTGGGGCG

CCCTCTGGTNAAGGTTGGGAAGCCCTGCAAAGTAAACTGGNTGGCTTTCTTGCCGCCAAGGATCTGATGGCGCNGGGNAT

CAAGCTCTGATCAAGAGACAGGATGAGGATCGTTTCGCATGATTGAACAAGATGGATTGCACGCAGNTNNTCCNGCNGCT

TGGGGNNNANNNGCTATTCGGCTATGACTNNNNNANNGANANCNGNTNNTCNNATNCNNNNNTNNNNNNCAGCGCNGGNN

NCCNNNNNNTTNNTCANACGACNNNCNNNNNCCNNNNNACNNNNANANNANGNNNNNNNNNNNNNNNNCNNNNNNNNNNN

NNNNNNNNNNNNNNNNNNNCNNNCNNNNNNNNNNNNNNNNNCNNNNNNN

Red text nucleotides represent the forward and reverse primer sequences. Grey shading represents the amplicon sequence.

**NCBI blast result:** No similarity

**Supplementary Figure 7.** Limited sequence identity was observed between the cloned insert and the intended target. This indicates that the gel band was a non-specific amplification product of similar electrophoretic mobility to the expected target. Red text nucleotides represent the forward and reverse primer sequences. Grey shading represents the amplicon sequence.
